# New Insights on the Impact of Cattle Handling on Post-Mortem Myofibrillar Muscle Proteome and Meat Tenderization

**DOI:** 10.3390/foods10123115

**Published:** 2021-12-15

**Authors:** Verónica Sierra, Laura González-Blanco, Yolanda Diñeiro, Fernando Díaz, María Josefa García-Espina, Ana Coto-Montes, Mohammed Gagaoua, Mamen Oliván

**Affiliations:** 1 Área de Sistemas de Producción Animal, Servicio Regional de Investigación y Desarrollo Agroalimentario (SERIDA), Ctra. AS-267, PK 19, 33300 Villaviciosa, Spain; veroniss@serida.org (V.S.); lgblanco@serida.org (L.G.-B.); ydineiro@serida.org (Y.D.); ferdm89@gmail.com (F.D.); mjgarcia@serida.org (M.J.G.-E.); 2 Instituto de Investigación Sanitaria del Principado de Asturias (ISPA), Av. del Hospital Universitario, s/n, 33011 Oviedo, Spain; acoto@uniovi.es; 3 Department of Morphology and Cell Biology, Faculty of Medicine, University of Oviedo, Av. Julián Clavería, 6, 33006 Oviedo, Spain; 4 Food Quality and Sensory Science Department, Teagasc Food Research Centre, Dublin 15, D15 KN3K Ashtown, Ireland

**Keywords:** intensive management, extensive management, mixing unfamiliar animals, myofibrillar proteins, pre-slaughter stress, protein biomarkers

## Abstract

This study investigated the effect of different cattle management strategies at farm (Intensive vs. Extensive) and during transport and lairage (mixing vs. non-mixing with unfamiliar animals) on the myofibrillar subproteome of *Longissimus thoracis* et *lumborum* (LTL) muscle of “Asturiana de los Valles” yearling bulls. It further aimed to study the relationships with beef quality traits including pH, color, and tenderness evaluated by Warner–Bratzler shear force (WBSF). Thus, comparative proteomics of the myofibrillar fraction along meat maturation (from 2 h to 14 days *post-mortem*) and different quality traits were analyzed. A total of 23 protein fragments corresponding to 21 unique proteins showed significant differences among the treatments (*p* < 0.05) due to any of the factors considered (Farm, Transport and Lairage, and *post-mortem* time ageing). The proteins belong to several biological pathways including three structural proteins (MYBPC2, TNNT3, and MYL1) and one metabolic enzyme (ALDOA) that were affected by both Farm and Transport/Lairage factors. ACTA1, LDB3, and FHL2 were affected by Farm factors, while TNNI2 and MYLPF (structural proteins), PKM (metabolic enzyme), and HSPB1 (small Heat shock protein) were affected by Transport/Lairage factors. Several correlations were found between the changing proteins (PKM, ALDOA, TNNI2, TNNT3, ACTA1, MYL1, and CRYAB) and color and tenderness beef quality traits, indicating their importance in the determination of meat quality and their possible use as putative biomarkers.

## 1. Introduction

Improving beef production and meat quality to cope with meet consumer demands is a major concern of the livestock production sector. It is well known that cattle intrinsic factors, such as breed and genetics, have a decisive influence on beef production and on the ultimate meat quality, therefore, different breeding strategies and meat maturation procedures must be adapted to the genetic diversity of the animals [1,2]. In this sense, there is great interest in promoting the development of native cattle breeds, as they seem to be more adapted to regional production systems and for promoting proximity trade as a sustainability strategy [3]. Apart from the intrinsic factors, there are also extrinsic factors, overall, from farm-to-fork, related to the routine handling of animals and animal-human interactions that must be considered to ensure beef quality [4]. Among them, production system and feeding strategies play an important role, not only due to the effect that dietary components may exert on the animal’s growth rate, and muscle/meat characteristics [2,5,6], but also due to their influence on the animal’s physiology, social behavior, and reactivity to stress [7,8,9,10]. Moreover, psychological, and physiological status of the animals can affect final meat quality [10]. In fact, cattle are herd animals that establish social orders, so the regrouping of animals or mixing with unfamiliar animals during transport and lairage, despite being a common husbandry practice, can have a detrimental effect on animal welfare, increasing animal stress [11,12].

On the other hand, animal handling may affect animal’s emotional state, hence inducing pre-slaughter stress (PSS), whose influence on the *post-mortem* process of muscle-to-meat conversion has been shown in pigs [13,14] and in cattle [3,9,10,15,16,17,18]. Those biochemical changes were evidenced using several high-throughput OMICs methods, including proteomics that revealed, for instance, that the meat tenderizing process involves myriad pathways, such as the degradation of structural proteins, energy metabolism pathways, response to stress, apoptosis, autophagy, and signaling pathways [19,20] as confirmed recently by the integromics meta-analysis study of Gagaoua et al. [21]. Comparative proteomics appeared to be a useful tool to study the biological pathways underpinning the effect of PSS on the ultimate meat quality. In this context, proteomic approaches point out the possible identification of putative biomarkers from the sarcoplasmic subproteome fraction of the *post-mortem* muscle [9,17,21,22]. Since tenderness and color are considered as important beef quality traits for consumers., the impact of PSS on these attributes is worthy of investigation. Indeed, PSS is proven to have a detrimental effect due to the changes that induces in the enzymatic processes that, for example, induce the breakdown of myofibrillar structure mainly composed of structural and contractile proteins [23,24,25,26].

Based on the above, this study aimed to apply a proteomics approach to investigate the effect of pre-slaughter factors such as mixing unfamiliar animals during the transport and lairage period on the myofibrillar subproteome of young “Asturiana de los Valles” bulls reared under two divergent rearing practices (intensive or extensive management systems). This trial further provides an opportunity to identify putative protein biomarkers [27] related to beef tenderization and PSS.

## 2. Materials and Methods

### 2.1. Animals and Experimental Design

This trial used 24 yearling bulls of “Asturiana de los Valles” (AV) breed that were slaughtered between 13 and 15 months of age. AV breed is a native breed from the north of Spain, with a high growth rate and low-fat content [28,29] and protected by the quality label “Ternera Asturiana”, which is one of the most significant in terms of production and economic value [30]. Calves were managed with their mothers from birth to weaning, fed on concentrate and barley straw ad libitum during the winter, and assigned in spring to two different farm management systems:

1) Intensive (“I”) (*n* = 12), with animals managed indoors, in pens of 6 × 6 m (6 animals per pen) and finished for 100 days before slaughter with 8 kg/day of concentrate (84% barley meal, 10% soya meal, 3% fat, 3% minerals, vitamins and oligoelements) and 2 kg/day of barley straw
2) Extensive (“E”) (*n* = 12), with animals managed outdoors in two 1.5 ha plots (6 animals per plot) and finished for 100 days before slaughter grazing on ryegrass and clover pasture + 3.5 kg/day of supplementation with concentrate.

At an approximate slaughter weight of 500 kg, the animals were transported in groups of six to a commercial abattoir located at around 40 km from the farm where the animals were finished. Half of them from each rearing system (I and E) was mixed with unfamiliar animals from other pens/groups not belonging to the study (mixing treatment “M”) and the other half (non-mixing treatment “NM”) was maintained in their original group for transport and lairage. Thus, there were six animals assigned to each group (I-M, I-NM, E-M, E-NM).

The experimental procedures were in compliance with the RD 53/201, where no authorization is required for practices carried out for recognized zootechnical purposes (Art 2.5d) and those that do not cause more pain than the introduction of a needle (Art 2.5f).

The pre-slaughter management lasted 6 h from when the animals left the farm, including the process of loading, travelling, unloading, and lairage, and was in accordance with the Council Regulation (EU) Nr. 1/2005, which relates to protecting the welfare of animals during transport and related operations. Animals were stunned with a captive bolt, slaughtered by immediate exsanguination, and dressed according to the current EU regulations (Council Regulation (EC) No 1099/2009) in accredited abattoirs. 

### 2.2. Muscle Sampling and Meat Quality Measurements

The carcasses were chilled at 3 °C within 2 h after slaughter. *Longissimus thoracis et lumborum* (LTL) muscle samples (20 g) were taken from the left-side carcass of each animal at the thirteenth rib level at 2 h, 8 h, and 24 h *post-mortem*. The muscle samples were immediately snap frozen in liquid nitrogen and stored at −80 °C until analysis.

At 24 h post-slaughter, the LTL muscle was removed from the left half carcass between the sixth and the tenth ribs, and transported to the laboratory. The LTL temperature and pH were recorded (pH24) at the sixth rib using a digital portable pH meter equipped with a penetration electrode coupled with a temperature probe (InLab Solids Go-ISM, Mettler-Toledo S.A.E., Barcelona, Spain).

Meat color was recorded at 24 h *post-mortem* on three 10 mm diameter spots on the exposed cut surface of the LTL muscle at the seventh rib level after 60 min blooming. The coordinates lightness (L*), redness (a*), and yellowness (b*) were obtained using a Minolta CM-2300d portable Spectrophotometer, with an illuminant C and D65 illuminant, 10° standard observer angle geometry and 8 mm aperture size in the CIE space (Konica Minolta Inc., Osaka, Japan), and the average value of the three spots was calculated. Further, both Chroma (C*) and Hue angle (h*) were calculated according to the next equations: C* = √ (a*^2^ + b*^2^) and h* = tan^−1^b*/a* [31]. 

The rest of the LTL striploin was sliced into 3.5 cm steaks that were vacuum packed in polyamide 20 μm/polyethylene 70 μm bags and aged in darkness under refrigerated conditions (4 °C ± 1 °C) at different *post-mortem* ageing times (3, 7, and 14 days). After the corresponding ageing period, steaks for comparative proteomics were frozen at −80 °C, while the steaks for meat toughness analysis were frozen and stored at −20 °C for subsequent analysis. Meat toughness was measured by the Warner–Bratzler (WB) shear test on meat cooked at 75 °C from 30 min by immersion in a water bath. After cooling, eight cores (1 cm^2^ in cross-section) from each steak were subjected to a perpendicular cut by the WB blade set HDP/WBV with a “V” slot using the TA.XT Plus instrument (Stable Micro Systems, London, UK). The maximum load (kg) required for total split was recorded, the results were subjected to detection of outliers by box plot and the extreme values were deleted. Results were expressed as the mean WB shear force maximum load for each steak. Tenderization rate (TR, %) was calculated as the percentage of decrease in WB shear force in a given period of time (3 to 7 days, 7 to 14 days, 3 to 14 days). 

### 2.3. Myofibrillar Protein Extraction

Proteomic analysis was performed on the muscle samples of the 24 animals. From each animal, muscle myofibrillar extracts were obtained at 2 h, 8 h, 24 h, 3 days, 7 days, and 14 days *post-mortem,* following the method described by Bjarnadottir et al. [32]. Briefly, 0.5 g muscle samples were homogenized in 4 mL of Tris-EDTA-Sucrose (TES) buffer containing 10 mM Tris [pH 7.6], 1 mM EDTA [pH 8.0], 0.25 M sucrose, and 0.6% protease inhibitor cocktail [P8340, Sigma-Aldrich Co., St. Louis, MO, USA], using a Polytron PT1200 E (Kinematica Inc., Luzern, Switzerland) two times for 15 s at maximum speed. The homogenate was centrifuged (20 min at 20,000× *g*) at 4° C. The resulting pellet was homogenized into 4 mL of lysis buffer containing 10 mM Tris-HCl pH [7.6], 7 M urea, 2 M thiourea, 2% CHAPS, and 10 mM DTT with the polytron 2 × 15 s at maximum speed. Subsequently, the solution was stirred at room temperature for 1 h in a Multi Reax stirrer (Heidolph Instruments, Schwabach, Germany) and was centrifuged at 20,000× *g* for 20 min at 4 °C. The supernatant containing the myofibrillar proteins was collected and filtered through a nylon filter (5 µm), aliquoted, and stored at −80 °C. The protein content of the extract was measured by the Bradford method [33].

### 2.4. Myofibrillar Subproteome Analysis (1D SDS-PAGE) and Protein Identification

The myofibrillar muscle extracts (30 µg) were prepared for SDS-PAGE as follows. First, they were denatured using a solution containing 65.8 mM Tris/HCl pH 6.8, 21% glycerol, 5% beta-mercaptoethanol, 2% SDS, 0.026% of bromophenol blue that were subsequently) heated at 100 °C for 5 min. Second, the denatured samples were loaded into 1 mm dual vertical slab gels (Mini-protean, Bio-Rad Laboratories Inc., Hercules, CA, USA) for separation using a 12% resolving gel and 4% stacking gel. Pre-stained molecular weight standards (Precision Plus Protein™ All Blue Standards, Bio-Rad Laboratories Inc., Hercules, CA, USA) were added on each gel.

Overall, three gels per sample were performed. The stained gel images were captured using the UMAX ImageScanner (Amersham Biosciences, Buckinghamshire, UK). The densitometry analysis and band quantification were carried out using Image Studio Lite 5.2.5 program (LI-COR Biosciences, Lincoln, NE, USA). To account for slight variations in protein loading, the optical density of protein bands was expressed as relative abundance (normalized volume) and expressed in arbitrary units. 

Bands of interest (with significant differences among the groups) were manually excised from the gels and prepared for identification by MALDI-TOF/TOF mass spectrometry. The details of this procedure were previously described by Díaz et al. [9]. 

### 2.5. Statistical and Bioinformatics Analyses

Raw data were scrutinized for data entry errors and outliers by boxplot. Normality of variables was tested by a Kolmogorov–Smirnoff test. For meat quality traits (pH, meat color and WBSF), the effect of animal management at Farm “F” (I vs. E), during the Transport and Lairage “TL” (M vs. NM) and the interaction (F × TL) were analyzed using a General Lineal model procedure (SPSSv22.0 SPSS Inc., Chicago, IL, USA). Further a repeated measure ANOVA was used to investigate the effect of Treatment (I-M, I-NM, E-M, E-NM) on WBSF measured at 3, 7, and 14 days *post-mortem*.

For myofibrillar bands intensities measured at different *post-mortem* times (2 h, 8 h, 24 h, 3 days, 7 days, and 14 days), the ANOVA model included Farm (F), Transport and Lairage (TL), ageing time (t), and their interactions as fixed factors and animal as covariate. 

Significant differences among *post-mortem* times were studied by the Tukey’s post-hoc test (Games–Howell when the variances were not homogeneous) at a significant level of *p* ≤ 0.05.

The relationships between meat quality traits and the myofibrillar subproteome at different *post-mortem* times were calculated by bivariate Pearson’s correlations. Moreover, principal component analysis (PCA) was performed to study the relationships among the meat quality traits and the differential proteins along meat tenderization. 

The significantly changing protein bands (differential proteins) were investigated using Metascape open-source tool to identify the main enriched Gene Ontology (GO) terms among the proteins following the procedures described by Gagaoua et al. [22,34]. The STRING database (Search Tool for Retrieval of Interacting Genes, ver. 11.0 at https://string-db.org/, accessed on 10 October 2021) was further used to construct the Protein–Protein Interactions (PPI) relating the differential proteins according to the pathways to which they belong. Moreover, the list of the identified proteins that differ among the groups were compared to the repertoire of Gagaoua et al. [21] to identify the extent of overlap with the previously identified beef tenderness biomarkers in LTL muscle.

## 3. Results

### 3.1. Meat Quality Attributes

The handling conditions (F and TL) had no significant effect on pH24 that showed normal values within the range 5.43–5.52, whatever the treatments. However, animals’ farm management (F) affected meat color parameters (Table 1), being L* (*p* < 0.05) b*, C*, and h* (*p* < 0.001,) significantly lower in meat from the extensive system, that was brownish and darker, which agrees with previous literature that describes that meat from grass-fed animals is darker than meat from grain-fed animals, and attributes these differences to diet, physical activity, or a combination of both [35,36,37]. 

The effect of animal mixing during transport and lairage (TL) and the F × TL interaction were significant for a* and C* (*p* < 0.05). In both parameters, meat from mixed animals had higher values compared to the meat from non-mixed animals, being a* (12.45 vs. 10.4) and C* (18.49 vs. 16.13) for M and NM, respectively. 

Farm rearing system were found to significantly affect meat toughness (*p* < 0.001) at 14 days *post-mortem* when meat from the intensive treatment showed lower values. These findings are in agreement to those of previous studies that found a negative effect of extensive treatment on tenderness [38,39].

**Figure 1:**
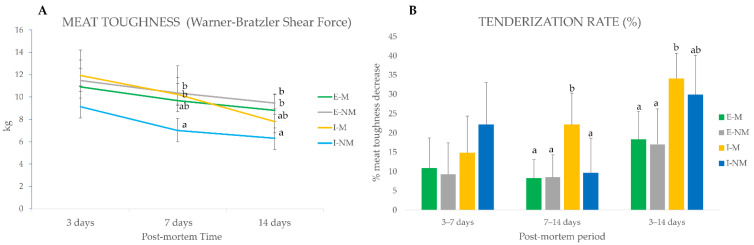
*Post-mortem* evolution of instrumental toughness for meat from the different handling treatments. (**A**) Warner–Braztler Shear force at 3, 7, and 14 days *post-mortem*. (**B**) Tenderization rate (%) calculated as the percentage of decrease in toughness in a given period of time (3 to 7 days, 7 to 14 days, 3 to 14 days). Different letters indicate significant differences (*p* < 0.05) among treatments (I-M, I-NM, E-M and E-NM) at 3, 7 or 14 days.

When comparing the evolution of meat toughness in the different handling treatments (I-M, I-NM, E-M and E-NM) (Figure 1A), no significant differences were found at 3 days *post-mortem*; however lower values of WBSF were found at 7 (*p* < 0.05) and 14 days (*p* < 0.001) in meat from the I-NM animals. When looking to the tenderization rate (Figure 1B), a decrease in meat toughness along ageing, it can be seen that the meat from animals of the intensive treatment had a higher tenderization rate in the global studied period (3 to 14 days) being higher for I-NM animals (22%) in the earliest period from 3 to 7 days, and for I-M animals in the last period of the *post-mortem* ageing (22%) from 7 to 14 days.

Overall, meat from animals of the I-M group showed redder meat and a lower tenderization rate, which could together be related to higher PSS. In fact, previous studies of the serum biomarkers of stress (cortisol, lactate, glucose, amyloid A, and haptoglobin) in this group of animals [9,18] evidenced that I-M animals were the most sensitive to stress reactivity, as indicated by its high serum haptoglobin levels.

### 3.2. Separation and Identification of Myofibrillar Subproteome

**Figure 2:**
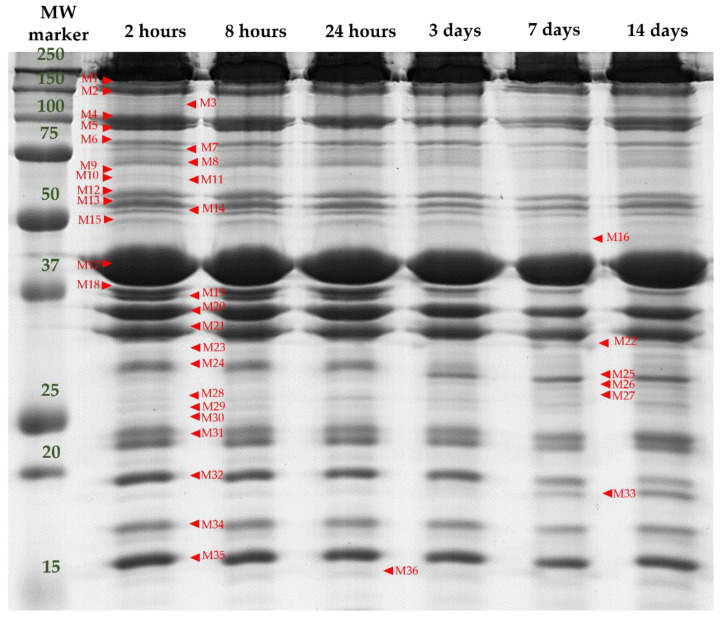
A representative 1D SDS-PAGE electrophoretic pattern of the myofibrillar subproteome profile of the LTL muscle of a yearling bull from “Asturiana de los Valles” at different *post-mortem* times (2 h, 8 h, 24 h, 3 days, 7 days, 14 days). MW marker: pre-stained molecular weight marker (All Blue pre-stained, Biorad). Band names are denoted by M (Myofibrillar proteins) followed by a number.

1D SDS-PAGE of the myofibrillar proteins allowed the visualization of 36 protein bands (ranging from 15 to 200 kDa) from the muscle myofibrillar subproteome, as shown in Figure 2.

Among the 36 bands analyzed in the myofibrillar subproteome, 23 bands were significantly affected by at least one of the factors analyzed in this study (F, TL, and t), as shown in Table 2.

It is important to note that these band intensity differences are due to the effect that handling factors may have in either the synthesis of a determined protein and/or to variations in the muscle *post-mortem* metabolism, which may result in increased proteolysis of that protein decreasing its relative abundance or causing its disappearance and the consequent increases of smaller protein fragments/peptides. Therefore, the relative abundance of a given (intact) protein is a balance between synthesis and degradation [22].

**Figure 3:**
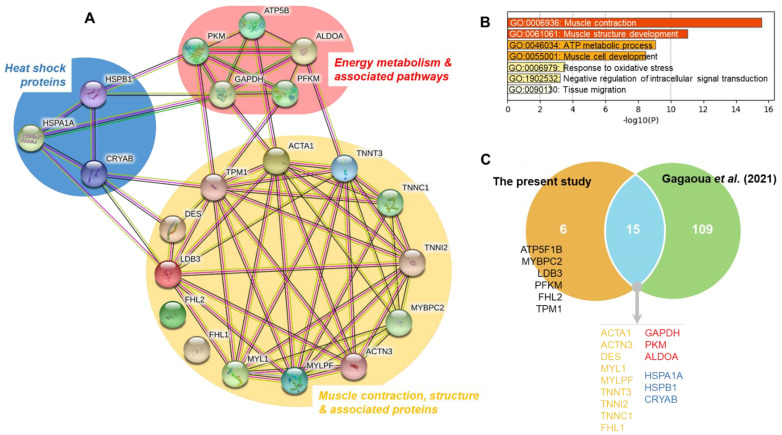
Bioinformatics analyses of the differential proteins affected with the treatments (Farm, Transport and Lairage and/or *post-mortem* time). (**A**) Protein-Protein Interactions of the 21 proteins using String database, highlighting 3 major pathways these being Muscle contraction, structure & associated proteins, Energy metabolism & associated pathways and Heat Shock proteins. (**B**) Significant enriched Gene Ontology (GO) terms obtained using Metascape tool. (**C**) Overlap among the 21 proteins with the repertoire of beef tenderness biomarkers reported in the database of Gagaoua et al. [18]. The 15 common proteins were highlighted by the corresponding molecular pathway color as in (**A**).

Table 3 shows the identification of protein bands with significant intensity differences among treatments. These proteins belong to three major biological pathways (Figure 3A):

➢ Muscle contraction, structure and associated proteins: M1 (Myosin-binding protein C, fast-type isoform X2 “MYBPC2”), M4 (Alpha-actinin-3 “ACTN3”), M13 (Desmin, partial “DES”), M17 (Actin, alpha skeletal muscle “ACTA1”), M21 (Tropomyosin alpha-1 chain “TPM1”), M23 (LIM domain-binding protein 3 isoform X5 “LDB3”), M24 (Four and a half LIM domains protein 1 isoform 1 “FHL1”), M25 and M27 (Troponin T, fast skeletal muscle isoform X31 “TNNT3”), M26 (Four and a half LIM domains protein 1 isoform 2 “FHL2”), M31 (Myosin light chain 1/3 skeletal muscle isoform “MYL1”), M32 (Troponin I, fast skeletal muscle “TNNI2”), M34 (Troponin C, skeletal muscle “TNNC1” and M35 (Myosin regulatory light chain 2, skeletal muscle isoform “MYLPF”);
➢ Energy metabolism and associated pathways: M6 (ATP-dependent 6-phosphofructokinase, muscle type “PFKM”), M12 (Pyruvate kinase PKM isoform X1 “PKM”), M15 (ATP synthase subunit beta, mitochondrial precursor “ATP5F1B”), M18 y M19 (Fructose-biphosphate aldolase A “ALDOA”) and M20 (Glyceraldehide-3-phosphate dehydrogenase, “GAPDH”);
➢ Heat shock proteins: M9 (Heat Shock 70 kDa protein 1A “HSPA1A”), M30 (Heat Shock protein family B member 1 variant 1 “HSPB1”) and M33 (Alpha-crystallin B chain “CRYAB”).

It is important to note that some proteins appeared in more than one band, as was the case of ALDOA (M18 and M19) and TNNT3 (M25 and M27), probably due to differences in the molecular weight or to conformational changes of the different proteolytic fragments originated from the same protein. The analyses of these differential proteins, which finally constitute 21 unique proteins (Table 3 and Figure 3A), allowed for the construction of an interconnected network (Figure 3A), highlighting the importance of muscle contraction and structure pathways. The Gene Ontology (GO) analysis allowed for the identification of seven enriched GO terms (Figure 3B), from which the top two enriched terms being GO:0006936: Muscle contraction and GO:0061061: Muscle structure development. These were followed by GO:0046034: ATP metabolic process, GO:0055001: Muscle cell development, GO:0006979: Response to oxidative stress, GO:1902532: Negative regulation of intracellular signal transduction and GO:0090130: Tissue migration.

It must be noted that most protein bands from the myofibrillar subproteome (61%) correspond to structural insoluble proteins, but soluble proteins such as glycolytic enzymes (26%) or HSPs (13%) were also found in the myofibrillar fraction (Figure 3A). This can be due to a decrease of solubility, maybe partly as a consequence of the early pH drop while the muscle temperature is still high, and also to the relationships that exist among the proteins [34], as confirmed in the network of Figure 3A. In fact, such conditions may cause denaturation of proteins which became insoluble, and their aggregation and precipitation onto myofibrils [24,40]. In support of this, the recent study by Gagaoua et al. showed that the maturation process involves interconnected molecular pathways in a pH-dependent manner leading, for instance, to the concomitant appearance of two major proteolytic fragments at 110 and 30 kDa, based on 1DE electrophoresis [34]. These two protein bands appearing during ageing, also observed in this study, increase in their intensity as a function of *post-mortem* time in a pH decline-dependent manner. LC-MS/MS analysis yielded 22 unique proteins for the 110 kDa fragment and 13 for the 30 kDa, with four common proteins related to both actin and fibrinogen complex. The Gene Ontology analysis revealed that a myriad of biological pathways are influential with many of them, as confirmed in the present study (Figure 3), and were related to proteins involved primarily in muscle contraction and structure. Other pathways were apoptotic mitochondrial changes, calcium and ion transport, energy metabolism, etc. Interestingly, most of the proteins composing these two fragments among others that appear or disappear during the tenderization process and in line to the results of this study have been so far identified as biomarkers of beef tenderness (Gagaoua et al., 2021) [21]. In addition, HSPs can translocate and accumulate in the cytoskeleton and myofibrillar proteins during early *post-mortem* stages, as they exert a protective role against muscle degradation [40]. These facts reinforce the need to consider different cell fractions and the movements of proteins between cytoskeletal and myofibrillar structures, for an accurate and reliable study of the process of conversion of the muscle into meat, as has been highlighted by previous studies [21,41].

#### 3.2.1. Handling Effects on the Muscle Contraction, Structure and Associated Proteins 

Among the protein bands with structural and contraction functions, only M27 (27.25 kDa), identified as Troponin T, fast skeletal muscle isoform X31 (TNNT3), was affected by the three factors analyzed in this study (F, TL, and t). Higher intensities of M27 were found in the muscle of the animals from the extensive treatment (*p* < 0.01) and mixed group animals during transport and lairage (*p* < 0.05). Further, a significant increase (*p* < 0.001) of TNNT3 band intensity with *post-mortem* time was found. Band M25 with a molecular weight of 28.67 kDa, was also identified as TNNT3, but it did not show significant differences with F or TL, but a significant increase with *post-mortem* time (*p* < 0.001). Troponin T is the most frequently identified differential biomarker of ongoing proteolysis and tenderization due to the appearance of degradation fragments of 30 kDa and 28 kDa correlated to meat tenderness [25,34,42,43,44]. TNNT3 is one of the proteins of the Troponin complex, composed of three regulatory proteins (Troponin T, C and I) that are integral to muscle contraction. It is well known that tenderization acts on all the proteins of the complex and it was identified as a robust biomarker of beef tenderness in the study of Gagaoua et al. [21], as evidenced in the Venn diagram of Figure 3C. Accordingly, our results showed changes in all the proteins of the Troponin complex. Bands M32 and M34 were identified as TNNI2 and TNNC1 respectively. TNNI2 was affected by TL, with higher intensity (*p* < 0.01) in meat from the mixed animals, and also a significant effect of *post-mortem* time was observed with a significant decrease (*p* < 0.001) after 3 days *post-mortem* in meat from E-M, E-NM and I-NM treatments, but delayed for I-M, which seems to indicate slower tenderization rate. 

Other bands with significant changes correspond to myosin related proteins M1 (Myosin-binding protein C, fast-type isoform X2 “MYBPC2”) and M31 (Myosin light chain 1/3 skeletal muscle isoform “MYL1”) were affected by both F and TL but not by ageing time. MYBPC2 showed higher intensity (*p* < 0.05) in meat from the indoor reared animals and in meat from the non-mixed group (*p* < 0.01), which could be related to differences in the level of physical exercise, as found for MYOM2, a major component of the myofibrillar subproteome that appeared in the sarcoplasmic subproteome of these animals, in agreement with the recent studies of Diaz et al., [9] and Gagaoua et al. [34]. MYBPC2 belongs to the Myosin Binding Proteins family formed by sarcomeric proteins located in the A-band in close association with the thick filaments that are known as regulators of the myofilament contractility [45]. MYBPC exists in three main isoforms: skeletal slow (MYBPC1), skeletal fast (MYBPC2), and cardiac (MYBPC3). MYPBC1 and MYBPC2 were recently identified to be the major components of the 110 kDa fragment appearing during the tenderization of beef in a pH dependent manner [34]. It is also important to note that a closely member, the myosin binding protein H (MYBPH) has been previously identified as a negative biomarker of color and beef tenderness, whatever the gender, due to its significant effect on length, thickness, and lateral alignment of myosin filaments [21,46,47]. In this work, MYBPC2 showed higher intensity levels in the I-NM meat, which was the tenderer one, hence confirming the findings by Gagaoua et al. [21,34] proposing this protein as a good marker of meat tenderization. 

MYBPC has a theoretical molecular weight of approximately 130 kDa; however, the band identified as MYBPC2 in our study shows a higher experimental molecular weight (155 kDa) what could be indicative of aggregation of this protein or to its interaction with nebulin and other proteins as observed in earlier studies [34,48]. These modifications may explain the loss of its function in the alignment of myosin filaments, and therefore the positive role it might play on tenderness. On the other hand, MYL1 is a member of the Myosin light chains that are crucial for muscle function in terms of contraction velocity and power. In this work, MYL1 band showed higher intensities in the meat reared outdoors (*p* < 0.05) and in meat from mixed animals (*p* < 0.01). Apart from MYL1, another band (M35) from the same family was identified as Myosin regulatory light chain 2, skeletal muscle isoform “MYLPF” that was not different within farm management, but was significantly affected by TL with higher values at the M treatment (*p* < 0.05) in line with the findings for MYL1. *Post-mortem* disruption of myosin light and heavy chains and actin in the actomyosin complex plays a central role in the muscle to meat conversion and may have a direct effect on tenderness [49]. The *post-mortem* concentration of these proteins has been previously associated with pork and beef tenderness [50,51], and belong to the robust list of biomarkers of beef tenderness shortlisted by Gagaoua et al. [21]. Increased proteolysis of MYL1 has also been described in dark-cutting beef [22,26]. It is worthy to note that myosin lights chains were also identified as biomarkers of several beef color traits [47].

Apart from the aforementioned, management at farms affected other three structural proteins: M17 (Actin, alpha skeletal muscle “ACTA1”), M23 (LIM domain-binding protein 3 isoform X5 “LDB3) and M26 (Four and a half LIM domains protein 1 isoform 2 “FHL2”). All of them were more intense (*p* < 0.05) in the meat from animals reared outdoors. Actin is the second most abundant myofibrillar protein, after myosin, and was described as the top biomarker of beef tenderness (Figure 3C) [21]. The breakdown of transverse cytoskeletal actin filaments can cause detachment of the sarcolemma from the basal lamina and the extracellular matrix network, causing muscle cells degradation and hence increasing tenderness; therefore, actin has been found to be a good biomarker of tenderness [21,40,49,52]. The other two proteins affected by farm management were the LIM-domain containing proteins, LDB3 and FHL2. The LIM domain is a cysteine-histidine rich, zinc-coordinating domain, consisting of two tandemly repeated zinc fingers. The LIM domain-containing proteins are known to play critical roles in vertebrate development and cellular differentiation. The LDB3 protein, located in the sarcomere, is essential for maintaining Z line structure and muscle integrity [53]. FHL2 is a member of the four and a half LIM domain protein family (FHL), with an important role in muscle development [54]. To the best of our knowledge, this protein has never been related to meat quality before; however another protein from the FHL family, the Four and a half LIM domains protein 1 isoform 1 (FHL1,) also known as Cypher protein, has been related to the release of α-actinin and the weakening of the Z-disc during meat tenderization [55,56]. In the present study, band M24 was identified as FHL1 and, contrary to what was found for FHL2, only a significant decrease with *post-mortem* ageing and a significant interaction F × TL (E-NM > E-M = I-M > I-NM) was observed (*p* < 0.001). Previous studies found increased intensity of FHL1 in DFD meat [16,22,57].

The other structural protein bands found in the myofibrillar extract were affected only by *post-mortem* time, with a significant decrease (*p* < 0.01) of M4 (Alpha-actinin-3 “ACTN3”) and M13 (Desmin, partial “DES”) and a significant increase (*p* < 0.001) of M21 (Tropomyosin alpha-1 chain “TPM1”). It is important to note that the *post-mortem* ageing time is the factor that causes the greatest differences in the intensity of the muscle contraction and structural proteins, with 10 out of the 14 structural bands showing significant differences in agreement to the very recent findings of Gagaoua et al. [34]. Most of these band’s intensities remain similar at the earliest *post-mortem* period (from 0 h to 24 h), but then increase or decrease drastically as a consequence of proteolysis. 

#### 3.2.2. Handling Effects on the Energy Metabolism and Associated Pathways Proteins

Most of the bands related to energy metabolism found in the myofibrillar fraction were glycolytic enzymes (Figure 3), usually found in the sarcoplasmic fraction. Among them, only the band M19 (36.88 kDa), corresponding to Fructose-biphosphate aldolase A “ALDOA”, was affected by the three factors studied: F, TL, and ageing time, showing higher intensity in meat from the Intensive rearing system (*p* < 0.01) and from the NM animals (*p* < 0.01), and a significant decrease along *post-mortem* ageing (*p* < 0.001) that starts at 8 h *post-mortem* in the meat from the Intensive treatment and at 24 h from the Extensive system (significant interaction (*p* < 0.01) F × t). Band M18 (37.64 kDa), also identified as ALDOA, showed a significant *post-mortem* decrease. In contrast with these results in the myofibrillar fraction, previous studies in the same animals showed higher intensity of ALDOA (37.1 kDa) in the sarcoplasmic subproteome of meat from the extensive reared animals [9]. ALDOA catalyzes the conversion of fructose 1, 6-diphosphate to glyceraldehyde 3-phosphate during glycolysis, therefore its lower intensity in meat from Intensive reared animals in the sarcoplasmic fraction can be associated with a faster glycogenolysis exhaustion or degradation of the enzyme in these animals that were found to be more susceptible to pre-slaughter stress [9]. However, it is also known that ALDOA in association with other metabolic enzymes, assists in the creation of cross-links between adjacent actin filaments or in binding troponin to the thin filaments, to enhance energy provision, where it is actively needed during contraction, hence affecting the distance between myofibrils, and therefore light scattering and tenderness [57]. This could at least partially explain the differences found between sarcoplasmic and myofibrillar ALDOA contents, as, due to the higher glycolytic metabolism of Intensive reared animals at slaughter, more energy provision may be needed for contraction and more ALDOA can be retained within the interstitial spaces of the myofibrils during the extraction. It is worthy to note that ALDOA was identified as a robust biomarker of beef tenderness (Figure 3C) [18] and of color variation [47]. 

Apart from ALDOA, Farm management did not affect significantly any of the other proteins from the energy metabolic pathway, however TL affected significantly to the band M12 (Pyruvate kinase PKM isoform X1 “PKM”) with increased intensity (*p* < 0.01) in the meat from the NM group under a significant F × TL interaction (*p* < 0.001). This band was the only one corresponding to metabolic enzymes that did not show significant differences with *post-mortem* time. PKM is a glycolytic enzyme implicated in the last phases of the glycolysis that catalyzes the dephosphorylation of phosphoenolpyruvate to pyruvate, yielding one molecule of pyruvic acid and one of ATP. Previous studies have shown that animals mixed during transport and lairage are more affected by pre-slaughter stress and they have also been related to higher *post-mortem* glycolytic metabolism [9,58]. Moreover, lower abundance of PKM was previously found in myofibrillar subproteome of high pH24 meat [26]. Thus, the lower levels of PKM found in the myofibrillar fraction of mixed animals in the present study can be explained by the glycogen depletion occurring due to PSS caused by the mixing procedure before slaughter. This can lead to a decrease in the glycolysis rate after slaughter due to the early depletion of glycogen and a downregulation of proteins involved in the glycolytic pathway in conjunction with oxidative pathways driven by mitochondria (for review see Gagaoua et al. [22]). 

The other metabolic enzymes, PFKM, ATP5F1B, and GAPDH, showed significant differences (*p* < 0.01) only with *post-mortem* time, with increasing band intensities during the early *post-mortem* until 24 h or 3 days *post-mortem* and decreasing afterwards. This reflects the cell metabolism behavior, with high levels of this proteins at early *post-mortem* due to the trigger of the glycolytic metabolism but decreasing later due to the impact of pH that might desaturate them or significantly reduce their activity. 

#### 3.2.3. Handling Effects on the Heat Shock Proteins

Overall, three bands from the myofibrillar subproteome were identified as members of the Heat Shock proteins (HSPs) family: M9 was identified as the large Heat shock 70 kDa protein 1A “HSPA1A”, and the other two (M30 and M33) as members of the small HSPs subfamily, identified as Heat Shock protein family B member 1 variant 1 “HSPB1” (also known as HSP27) and Alpha-crystallin B chain “CRYAB”. Functional proteomic studies have confirmed differential expression of HSPs in meat from different breeds, handling systems and quality traits [16,21,32,47,59,60]. In the present study, all the HSPs bands showed significant changes along the *post-mortem* meat maturation, with a significant increase (*p* < 0.01) up to 24 h *post-mortem* followed by a progressive decrease afterwards in the case of HSPB1, a significant increase after three days (*p* < 0.001) for HSPA1A and a significant decrease (*p* < 0.001) after three days for CRYAB. Surprisingly, none of these bands were affected by management at farm, and the mixing treatment affected significantly only HSPB1 band that was more intense (*p* < 0.001) in meat from the non-Mixed group. Moreover, a significant interaction F × TL (*p* < 0.001) was found for this band. 

Among the small HSPs, HSPB1 is one of the most frequently related protein to beef tenderness but in different directions (positive or negative manner) depending on the studied factors (PSS, breed, gender, post-translational modifications (isoform), rearing factor, etc.), as explained by Gagaoua et al. [21]. HSPs exert protective functions as chaperone proteins from proteases, therefore they could reduce degradation of myofibrillar proteins [61]. Several studies reported positive relationship between degradation of HSPB1 and meat tenderness improvements, as degraded HSPs may no longer prevent irreversible damage to myofibrillar proteins [1,60,62,63]. Accordingly, our results showed higher intensity of HSPB1 band in the myofibrillar subproteome of meat from the E-NM treatment, which was the treatment that showed higher WBSF, and thus a lower tenderization rate. Moreover, the evolution of HSPB1 along meat ageing is in agreement with previous studies in bovine muscles [50,64,65] that have demonstrated that muscle HSPB1 increases in abundance shortly after slaughter but decreases during meat storage. Similarly, our results showed a significant decrease (*p* < 0.001) of the large HSPA1A with *post-mortem* ageing. Members of the HSP70 family were previously found in the sarcoplasmic subproteome of these animals with a significant effect of Farm management, showing higher intensity in meat from the extensive reared animals [9]. Under stress situations, HSPA1A, which is an inducible protein that translocates and accumulates in the cytoskeletal and myofibrillar proteins in an attempt of stabilizing the muscle structure [40]. This HSP diffusion capacity between cellular fractions may explain the differences found between rearing treatments. As aforementioned, animals from intensive rearing system are suggested to be more susceptible to handling and pre-slaughter stress [9,18] so that more HSP may translocate to myofibrils while in the extensively reared group more HSPA-A is probably easily removed and extracted from the sarcoplasmic fraction. 

CRYAB was affected by *post-mortem* time showing a significant (*p* < 0.001) increase in intensity after seven days of storage. In contrast, previous studies demonstrate a decrease of intact CRYAB with ageing in total extracts from different muscles (Longissimus lumborum, Semimembranosus and Psoas major) of Angus x Simmental beef cattle [60]. Our results showing late *post-mortem* increases of this band could be explained by solubility changes due to protein modifications such as fragmentation, oxidation, precipitation, or aggregation thereby going from soluble to an insoluble state. This was previously pointed out by Bjarnadóttir et al. [32], who discovered that some of the small HSPs proteins increased their abundance in the insoluble protein fraction, possibly as a result of aggregation onto myofibrillar proteins, thereby following them during extraction. It is also important to note that CRYAB behaves also as a structural protein and therefore it can be susceptible of degradation along ageing. 

### 3.3. Relationship between Meat Quality Traits and the Significantly Changing Myofibrillar Proteins

The correlations between the differential protein bands from the myofibrillar subproteome and meat quality traits, measured at different *post-mortem* times were analyzed, and significant correlations with color traits and meat toughness are shown in Table 4 and Table 5 respectively. 

In the present study beef color traits were correlated (Table 4) with two metabolic enzymes (PKM and ALDOA), two Heat Shock proteins (HSPA1A and HSPB1) and three structural proteins (DES, TNNI2 and MYLPF). This confirms the knowledge about the importance of cell glycolytic rate and its consequences on *post-mortem* modifications of proteins such as myosin, actin, troponin, and other metabolic proteins, particularly glycolytic enzymes in the sarcoplasm, and therefore influences the ultimate meat color. 

A recent integromics study evidenced that both glycolytic enzymes and HSPs pathways have important roles in beef color determination where several relevant biomarkers were shortlisted [21]. Accordingly, PKM showed significant (*p* < 0.05) positive correlations with L*, b* and h* and negative with a* and C*. The highest correlation coefficients were found at 8 h *post-mortem* with a* (−0.850, *p* < 0.01) and h* (0.800, *p* < 0.01). ALDOA showed the highest correlations with a* and h* at 7 days *post-mortem* (0.821, *p* < 0.01 and −0.695, *p* < 0.05, respectively). Other correlations were found between color traits and GAPDH (0.701, *p* < 0.05 with a* and −0.705, *p* < 0.01 with h*) at 7 days *post-mortem*. PKM and ALDOA may exert their effect on muscle color due to their involvement in the glycolytic pathway providing energy to the muscle contraction. They can also assist in the creation of crosslinks between actin filaments or in binding troponin to the thin filaments, hence affecting the distance between myofibrils and therefore light scattering [21,22,66,67,68,69]. In the case of HSPs, we found positive correlations between HSPA1A and L* and h* (+0.731, *p* < 0.05 at 8 h *post-mortem*) and negative between HSPB1 and L* and b* (−0.73, *p* < 0.01 at 7 days *post-mortem*). Many studies have related HSPs with color [70,71,72] probably due to their protective action against stress-induced denaturation of muscle proteins, that would affect reflectance, light scattering, and myoglobin, hence influencing color parameters [57,73,74,75,76,77].

Finally, strong positive correlations were found between some structural proteins and color, such as between MYLPF with L* and h* at 2 h *post-mortem* (0.7, *p* < 0.01) and between TNNI2 and L* and h* at 3 days post-mortem (0.75, *p* < 0.01), while negative correlations were found between a* and DES at 2 h post-mortem (−0.683, *p* < 0.05) and MYLPF at 3 days post-mortem (−0.707, *p* < 0.01). Hughes et al. [76] found that meat color was not determined only by chromatic heme pigments, but also by the physical structure and achromatic light scattering properties of the muscle. Therefore, the effect of structural proteins in meat color is related to their denaturation and degradation during the *post-mortem* process that affect the protein density along the sarcomere, and therefore light scattering from the structural elements as evidenced in the recent integromics proteomics meta-analyses of beef color and dark-cutting beef by Gagaoua et al. [22,47]. The current insights as revealed by both proteomics and conventional biochemical studies were further recently discussed by Purslow et al. [77]. The authors stated that it is increasingly likely that omics techniques, including proteomics, will be used to discover more of the complex interactions between pathways behind the qualities of meat and their determination.

Regarding tenderness, a total of 18 bands showed significant correlations with WBSF and/or with meat tenderization rate (% of WBSF decrease from 3 days to 14 days (Table 5). ACTA1, TNNT3, MYL1, ALDOA, and CRYAB were correlated with tenderness. These proteins showed the highest correlations with WBSF during the first 24 h *post-mortem*, being negative in the case of ALDOA, mainly with WBSF 3 days and ALDOA measured at 24 h *post-mortem* (−0.549, *p* < 0.05), and positive in the case of TNNT3 and WBSF at 14 days *post-mortem* (0.625, *p* < 0.01). Thus, these findings support the previous knowledge of the importance of these proteins (Figure 3C) as early biomarkers of meat tenderization. In agreement with these results, ALDOA was positively associated with tenderness [18]. Finally, and with respect to tenderness, CRYAB, showed a positive correlation at 2 h *post-mortem* with the tenderization rate from 3 to 14 days *post-mortem* (0.592, *p* < 0.01). Increased CRYAB levels were associated with delayed myofibril degradation in beef with ultimate pH < 5.7 [61]. 

**Figure 4:**
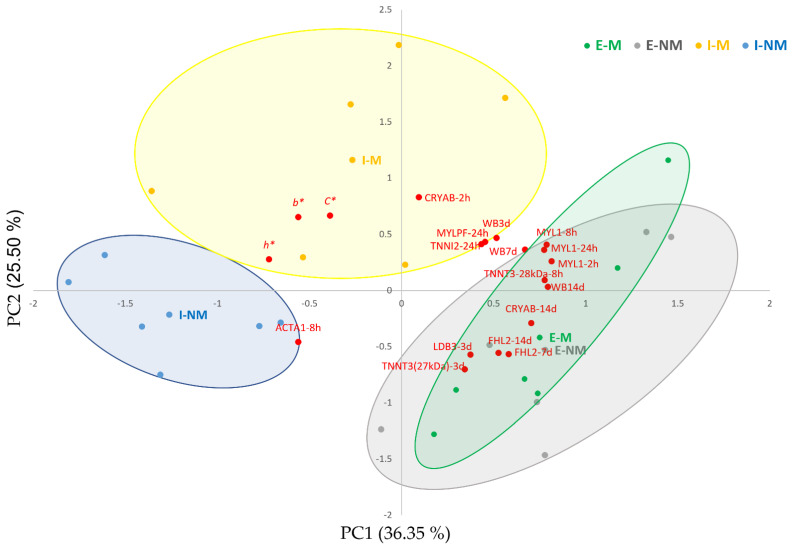
Biplot of variables and individuals (meat samples). The centroids of the animal treatments are shown in squares denoted with codes: I-M (Intensive-Mixed), I-NM (Intensive-Non-Mixed), E-M (Extensive Mixed), E-NM (Extensive-Non-Mixed). Individual samples are shown in yellow bullets (I-M), blue bullets (I-NM), green bullets (E-M) and grey bullets (E-NM). WB3 d: Warner–Bratzler Shear Force at 3 days, WB7 d: Warner–Bratzler Shear Force at 7 days, WB14 d: Warner–Bratzler Shear Force at 14 days; b*: yellowness; C*: Chroma; h*: hue angle; CRYAB: Alpha-crystallin B chain; MYL1: Myosin light chain 1/3 skeletal muscle isoform; MYLPF: Myosin regulatory light chain 2, skeletal muscle isoform; TNNT3: Troponin T, fast skeletal muscle isoform X31; TNNI2: Troponin I, fast skeletal muscle; LDB3: LIM domain-binding protein 3 isoform X5; FHL2: Four and a half LIM domains protein 1 isoform 2; ACTA1: Actin, alpha skeletal muscle. 2 h: 2 h *post-mortem*, 8 h: 8 h post-mortem; 24 h: 24 h *post-mortem*; 3 d: 3 days *post-mortem*; 7 d: 7 days *post-mortem*.

With the aim to summarize the complex relationships between meat quality traits and the myofibrillar subproteome, a PCA was performed including only the variables with higher correlation loadings (over 50% of explained variance). Figure 4 shows the biplot obtained by PCA between variables (loadings), individual meat samples (scores), and treatments (centroids). The first PC1 and PC2 explained 62% of the variability. The PC1 separated in the positive side the meat samples from the Extensive treatment, with higher meat WBSF values and overexpression of MYL1 at 2, 8, and 24 h, MYLPF at 24 h, LDB3 at 3 days, FHL2 at 7 and 14 days, TNNT3 (29 kDa) at 8 h, TNNT3 (27 kDa) at 3 days, TNNI2 at 24 h and CRYAB at 2 h. The meat samples from the Intensive treatment were located in the negative side, showing higher b* values, h* and C* and ACTA1 at 8 h *post-mortem*.

Multivariate analysis showed that the handling factors (F and TL) had a clear effect in the different variables analyzed. Thus, animal’s farm management produced a clear separation between meat samples from animals reared under Intensive or Extensive systems. On the other hand, the effect of Mixing animals during Transport and Lairage was significant in the intensively reared animals with a clear separation in two groups: (1) meat of the I-NM animals with lower WBSF values and higher intensity of the ACTA1 band at 8 h *post-mortem*; and (2) meat of the I-M animals with higher b*, h*, and C*. Our previous studies pointed out that these I-M animals suffered from higher PSS as they showed higher serum haptoglobin and glucose levels at slaughter and lower muscle ATP levels, thus resulting in the blockage of the muscle antioxidant defense and slower *post-mortem* autophagic rate [9,18]. In fact, response to oxidative stress was found as a significant enriched term in this study (Figure 3B), which need further studies in the future to better elucidate the underlying mechanisms about its role in relation to the factors we investigated in this study.

Within the extensively reared animals, the effect of social mixing was not very clear as mixed and non-mixed animals were overlapped in the positive side of the PC1, indicating a lower effect of mixing unfamiliar animals, as previously described [9]. It is difficult to determine if these differences between treatments are due to differences in diet, physical activity or higher PSS derived from the different animal’s handling treatments, but there are clear differences and the changes they produce in the myofibrillar subproteome at early *post-mortem* could provide putative biomarkers of the final meat quality. 

## 4. Conclusions

The findings of this study confirmed that meat quality of young “Asturiana de los Valles” bulls is affected by handling practices at Farm and during Transport and Lairage before slaughter and during the tenderization of the meat. At the farm level, the production system (Intensive vs. Extensive) significantly affected meat color parameters (L*, b*, C*, and h*), highlighting that the meat from the Extensive treatment was brownish and darker. The Transport and Lairage factor (Mixed vs. Non-Mixed) affected also color traits mainly redness (a*) and Chroma (C*) leading to lower values in non-mixed animals. The tenderization rate of the meats of the investigated groups was higher but delayed in the meat samples from I-M animals. The comparative proteomics extended our knowledge and revealed that Farm, Transport/Lairage and *post-mortem* ageing has huge but different effects on the *post-mortem* muscle myofibrillar subproteome. The major pathway that was impacted by this factor was related to muscle structure. In fact, farm management affected six structural proteins (MYBPC2, TNNT3, MYL1, ACTA1, LDB3, and FHL2) and one metabolic enzyme (ALDOA) while Transport and Lairage prior to slaughter induced changes in five structural protein bands (MYBPC2, TNNT3, TNNI2, MYL1, and MYLPF), two metabolic enzymes (PKM and ALDOA), and one Heat shock protein (HSPB1). 

*Post-mortem* ageing was the most important factor affecting the myofibrillar proteome, among the different handling practices confirming the importance of monitoring subproteome changes along meat ageing for an accurate understanding of the effects. Several correlations were found between the protein changing in this trial at early *post-mortem* times with meat color and tenderness parameters (PKM, ALDOA, HSPA1A, HSPB1, CRYAB, DES, TNNT3, TNNI2, and MYLPF), confirming that they could be used as meat quality biomarkers in comparison to the largest and recent beef tenderness biomarkers database recently published by Gagaoua et al. [21].

## Figures and Tables

**Table 1:** Effect of Farm (F) and Transport and Lairage (TL) and their interaction on meat color parameters.

	Farm Management (F)			Transport and Lairage (TL)			Significance		
Quality Traits	I	E	SEM	NM	M	SEM	F	TL	F × TL
pH	5.6	5.46	0.05	5.49	5.58	0.05	NS	NS	NS
L*	41.86	38.20	0.99	38.73	41.33	0.99	*	NS	NS
a*	12.03	10.82	0.523	10.4	12.45	0.523	NS	*	*
b*	15.49	10.28	0.522	12.19	13.58	0.522	***	NS	NS
C*	19.64	14.97	0.681	16.13	18.49	0.681	***	*	*
h*	52.49	43.61	1.04	49.07	47.03	1.04	***	NS	NS

F: Management at farm; TL: Management during transport and lairage; NM: Non-mixing; M: Mixing; NS: not significant; * *p* < 0.05; *** *p* < 0.001; SEM: standard error of the mean.

**Table 2:** Effect of handling factors (animal management at Farm (F) and during Transport and Lairage (TL)) and the *post-mortem* time (t) and their interactions (F **×** TL, F **×** t, TL **×** t and F **×** TL **×** t) on the myofibrillar subproteome (arbitrary units).

	Farm Management (F)			Transport and Lairage (TL)			Post-Mortem Time (t)								Significance					
Band [MWe]	I	E	SEM	NM	M	SEM	2 h	8 h	24 h	3 d	7 d	14 d	SEM	F	TL	t	F × TL	F × t	TL × t	F × TL× t
M1 (155.03)	4.66	3.67	0.25	4.76	3.87	0.15	4.08	4.39	4.22	4.4	4.36	3.57	0.24	*	**	NS	NS	NS	NS	NS
M4 (99.16)	5.94	4.97	0.29	5.48	5.43	0.17	6.04 b	5.99 b	5.71 ab	5.03ab	5.03ab	4.93 a	0.28	NS	NS	**	NS	NS	NS	NS
M6 (83.56)	2.18	2.0	0.09	2.102	2.08	0.06	2.05 ab	2.24 b	2.28 b	2.13 b	2.07 ab	1.78 a	0.09	NS	NS	**	NS	NS	NS	NS
M9 (71.02)	1.23	1.36	0.06	1.27	1.32	0.04	1.37 b	1.43 b	1.33 b	1.37 b	1.21 a	1.05 a	0.06	NS	NS	***	NS	NS	NS	NS
M12 (59.93)	2.63	2.22	0.15	2.63	2.22	0.09	2.35	2.53	2.61	2.60	2.35	2.12	0.15	NS	**	NS	***	NS	NS	NS
M13 (56.24)	2.42	2.49	0.09	2.47	2.44	0.05	2.47 ab	2.70 b	2.59 b	2.37 ab	2.37 ab	2.25 a	0.09	NS	NS	**	NS	NS	NS	NS
M15 (50.74)	1.5	1.57	0.06	1.49	1.58	0.04	1.47 ab	1.62 b	1.62 b	1.64 b	1.52 ab	1.34 a	0.06	NS	NS	**	*	NS	NS	NS
M17 (40.53)	16.14	19.3	0.79	17.64	17.8	0.47	19.19 ab	16.23 a	17.08 a	16.93 a	16.91 a	19.98 b	0.76	*	NS	**	NS	NS	NS	NS
M18 (37.64)	2.96	2.98	0.12	2.96	2.98	0.07	3.27 bc	3.49 c	3.38 c	2.82 ab	2.52 a	2.34 a	0.12	NS	NS	***	NS	NS	NS	NS
M19 (36.88)	2.34	1.94	0.07	2.24	2.05	0.04	2.61 c	2.73 c	2.67 c	1.99 b	1.49 a	1.38 a	0.07	**	**	***	NS	**	NS	NS
M20 (35.03)	7.47	7.04	0.22	7.28	7.23	0.13	7.09 abc	7.58 bc	8.02 c	7.68 b	6.84 ab	6.33 a	0.21	NS	NS	***	NS	NS	NS	NS
M21 (32.95)	6.1	5.9	0.17	6.01	5.99	0.1	5.26 a	5.7 ab	5.74 ab	6.56 c	6.62 c	6.1 c	0.16	NS	NS	***	NS	NS	NS	NS
M23 (30.89)	1.03	1.25	0.05	1.15	1.13	0.03	1.05 a	1.04 a	1.04 a	1.06 a	1.21 ab	1.44 b	0.05	*	NS	***	NS	NS	NS	NS
M24 (29.75)	2.4	2.6	0.1	2.49	2.51	0.06	3.04 d	2.89 d	2.74 cd	2.32 bc	2.17 ab	1.84 a	0.10	NS	NS	***	***	NS	NS	NS
M25 (28.67)	1.58	1.71	0.08	1.61	1.68	0.05	0.89 a	0.92 a	0.97 a	1.68 b	2.51 c	2.89 d	0.08	NS	NS	***	NS	*	NS	NS
M26 (27.82)	0.65	0.8	0.03	0.71	0.73	0.02	0.54 a	0.53 a	0.51 a	0.73 b	0.95 c	1.08 c	0.03	*	NS	***	*	*	NS	NS
M27 (27.25)	0.56	0.69	0.02	0.6	0.65	0.01	0.47 a	0.45 a	0.45 a	0.62 b	0.83 c	0.93 d	0.02	**	*	***	NS	NS	NS	NS
M30 (25.10)	1.22	1.33	0.06	1.35	1.2	0.04	1.33 bc	1.37 bc	1.42 c	1.24 abc	1.16 ab	1.11 a	0.06	NS	**	**	***	NS	NS	NS
M31 (23.20)	5.53	6.04	0.13	5.61	5.96	0.08	5.81	5.77	5.59	5.69	5.86	5.97	0.12	*	**	NS	NS	NS	NS	NS
M32 (19.89)	3.83	3.7	0.13	3.66	3.86	0.07	4.11 bc	4.09 bc	3.98 bc	4.23 c	3.64 b	2.52 a	0.12	NS	**	***	NS	***	NS	NS
M33 (19.07)	1.17	1.22	0.08	1.2	1.18	0.05	0.79 a	0.77 a	0.79 a	1.05 a	1.54 b	2.21 c	0.08	NS	NS	***	NS	***	NS	NS
M34 (17.54)	3.84	3.92	0.11	3.96	3.8	0.07	3.82	3.81	3.78	3.86	3.94	4.06	0.13	NS	NS	NS	*	NS	NS	NS
M35 (16.05)	5.76	5.37	0.14	5.43	5.7	0.08	5.45	5.49	5.34	5.62	5.73	5.76	0.05	NS	*	NS	NS	NS	NS	NS

Mwe: the experimental molecular weight (kDa), SEM: standard error of the mean; F: Management at farm; TL: Management during the transport and lairage; t: *Post-mortem* time; I: Intensive, E: Extensive; NM: Non-mixing with unfamiliar animals, M: Mixing with unfamiliar animals; Significance: NS: not significant; *: *p* < 0.001; **: *p* < 0.05; ***: *p* < 0.01. The variables not followed by the same letter in the same row (a, b, c, and d) are statistically different (*p* < 0.05).

**Table 3:** Protein identification of myofibrillar bands separated by 1D-SDS-PAGE that showed significant differences with treatments (Farm, Transport and Lairage, and/or *post-mortem* time).

Band: Gene Name	Identification	Accession Number	MOWSE Scores	Sequence Coverage (%)	Matched Queries	MWt
M1: MYBPC2	Myosin-binding protein C, fast-type isoform X2	E1BNV1	295	24	25	128.5
M4: ACTN3	Alpha-actinin-3	Q0III9	506	38	35	103.7
M6: PFKM	ATP-dependent 6-phosphofructokinase, muscle type	Q0IIG5	352	40	42	86.1
M9: HSPA1A	Heat Shock 70 kDa protein 1A	Q27975	288	38	24	70.5
M12: PKM	Pyruvate kinase PKM, isoform X1	A5D984	822	66	46	58.5
M13: DES	Desmin, partial	O62654	246	54	21	52.6
M15: ATP5F1B	ATP synthase subunit beta, mitochondrial precursor	P00829	445	48	25	56.2
M17: ACTA1	Actin, alpha skeletal muscle	P68138	522	52	24	42.4
M18: ALDOA	Fructose-biphosphate aldolase A	A6QLL8	430	62	24	39.9
M19: ALDOA	Fructose-biphosphate aldolase A	A6QLL8	286	60	21	39.9
M20: GAPDH	Glyceraldehide-3-phosphate dehydrogenase	P10096	394	45	20	36.1
M21: TPM1	Tropomyosin alpha-1 chain	Q5KR49	199	41	15	32.7
M23: LDB3	LIM domain-binding protein 3 isoform X5	G3N3C9	180	55	19	30.9
M24: FHL1	Four and a half LIM domains protein 1 isoform 1	G3MZ95	671	87	34	35.5
M25: TNNT3	Troponin T, fast skeletal muscle isoform X31	Q8MKI3	206	45	18	28.9
M26: FHL2	Four and a half LIM domains protein 1 isoform 2	Q2KI95	95	37	11	33.8
M27: TNNT3	Troponin T, fast skeletal muscle isoform X31	Q8MKI3	178	43	15	28.9
M30: HSPB1	Heat Shock protein family B member 1 variant 1	Q3T149	368	73	14	22.4
M31: MYL1	Myosin light chain 1/3 skeletal muscle isoform	A0JNJ5	425	77	18	21.1
M32: TNNI2	Troponin I, fast skeletal muscle	F6QIC1	194	70	23	21.6
M33: CRYAB	Alpha-crystallin B chain	P02510	163	69	13	20.1
M34: TNNC1	Troponin C, skeletal muscle	P63315	333	56	15	18.3
M35: MYLPF	Myosin regulatory light chain 2, skeletal muscle isoform	Q0P571	517	66	21	19.11

The MOWSE score: numeric descriptor of the likelihood that the identification is correct. Protein scores greater than 94 are significant (*p* < 0.05); Mwt: theoretical molecular weight (kDa).

**Table 4:** Significant Pearson correlations coefficients between myofibrillar subproteome bands and meat color traits.

		PFKM	HSPA1A	PKM	DES	ALDOA M18	ALDOA M19	GAPDH	TPM1	LDB3	FHL1	TNNT3 M25	TNNT3 M27	HSPB1	TNNI2	CRYAB	MYLPF
L*(60)	2 h		0.691 *							−0.654 *				−0.651 *	0.624 *		0.702 *
	8 h		0.731 **	0.613 *	0.608 *									−0.581 *	0.604 *		
	24 h				0.615 *										0.697 *		
	3 d													−0.687 *	0.768 **	−0.630*	0.610 *
	7 d							−0.652 *			−0.588 *	0.583 *		−0.777 **			.578 *
	14 d				0.599 *		.633*					0.615 *					
a*(60)	2 h			−0.695 *	−0.683 *												−0.593 *
	8 h	−0.588 *		−0.850 **													
	24 h	−0.694 *		−0.675 *													
	3 d			−0.726 *		0.685 *									−0.581 *		−0.707 *
	7 d					0.821 **		0.701 *				−0.592 *	−0.686*				−0.611 *
	14 d			−0.585 *								−0.632 *					
b*(60)	2 h		0.645 *							−0.633 *							0.608 *
	8 h		0.612 *						0.606 *						0.627 *		
	24 h				0.588 *										0.683 *		
	3 d									−0.664 *				−0.624 *	0.634 *	−0.597 *	
	7 d			0.581*							−0.650 *			−0.738 **			
	14 d																
C*(60)	2 h			−0.605 *	−0.584 *		−0.592 *										
	8 h			−0.613 *			−0.644 *										
	24 h	−0.714 *					−0.599 *										
	3 d			−0.637 *		0.651 *											
	7 d					0.666 *							−0.634*				
	14 d																
h* (60)	2 h		0.592 *		0.588 *					−0.617 *				−0.599 *			0.741 **
	8 h		0.700 *	0.800 **													
	24 h			0.670 *											0.610 *		0.578 *
	3 d			0.676 *											0.755 **		0.721 **
	7 d					−0.695 *		−0.751 **				0.626 *		−0.641 *			0.654 *
	14 d				0.689 *							0.618 *					

* *p* < 0.05; ** *p* < 0.01; PFKM: ATP-dependent 6-phosphofructokinase; HSPA1A: Heat shock 70 kDa protein 1A; PKM: Pyruvate kinase muscle; DES: Desmin; ALDOA: Fructose-biphosphate aldolase A; GAPDH: Glyceraldehide3-phosphate dehydrogenase; TPM1: Tropomyosin alpha 1; LDB3: LIM domain-binding protein 3; FHL1: Four and a half LIM domains protein 1 isoform 1; TNNT3: Troponin T fast skeletal muscle isoform x31; HSPB1: Heat shock protein family B member 1 variant 1; TNNI2: Troponin I fast skeletal muscle; CRYAB: Alpha-crystallin B chain; MYLPF: Myosin regulatory light chain 2. The higher and significant correlation coefficients are in bold.

**Table 5:** Significant Pearson correlations coefficients between myofibrillar subproteome bands and tenderness evaluated by Warner–Bratzler shear force and tenderization rate (%).

		ACTN3	HSPA1A	PKM	DES	ATP5F1B	ACTA1	ALDOA M18	ALDOA M19	GAPDH	FHL1	TNNT3 M25	FHL2	TNNT3 M27	MYL1	TNNI2	CRYAB	TNNC1
WB3 d	2 h			−0.482 *	−0.427 *				−0.437 *			0.539 **			0.415 *	0.497 *	0.423 *	
	8 h								−0.493 *						0.439 *			
	24 h		0.415 *			0.472 *			−0.545 **								0.428 *	
	3 d	0.495 *																
	7 d											−0.450 *						
	14 d						−0.521 **					−0.417 *						
WB7 d	2 h											0.556 **			0.503 *			
	8 h						−0.411 *					0.529 **			0.516 **			
	24 h								−0.502 *						0.453 *			
	3 d														0.472 *			
	7 d											−0.451 *						
	14 d	0.447 *					−0.599 **				0.413 *						0.514 *	
WB14 d	2 h								−0.441 *			0.514 *			0.483 *			0.417 *
	8 h								−0.524 **			0.625 **			0.503 *	0.405 *		0.441 *
	24 h								−0.421 *					0.445 *	0.456 *			
	3 d														0.542 **			
	7 d																0.521 *	
	14 d						−0.567 **				0.429 *						0.614 **	
% TR. 3–14 d	2 h																0.592 **	
	8 h																	
	24 h													−0.505 *				
	3 d							0.442 *					−0.491 *	−0.564 **				
	7 d												−0.546 **	−0.538 **				
	14 d									0.453 *			−0.557 **	−0.469 *				

* *p* < 0.05; ** *p* < 0.01. WB3 d: Warner–Braztler shear force 3 days; WB7 d: Warner–Braztler shear force 7 days; WB14 d: Warner–Braztler shear force 14 days; % TR. 3–14 d: Tenderization rate from 3 to 14 days *post-mortem*. ACTN3: Alpha A-actinin-3; HSPA1A: Heat Shock 70 kDa protein 1A; PKM: Pyruvate kinase muscle; DES: Desmin; ATP5F1B: ATP synthase subunit beta, mitochondrial precursor; ACTA1: Actin, alpha skeletal muscle; ALDOA: Fructose-biphosphate aldolase A; GAPDH: Glyceraldehide3-phosphate dehydrogenase; FHL1: Four and a half LIM domains protein 1 isoform 1; TNNT3: Troponin T fast skeletal muscle isoform x31; FHL-2: Four and a half LIM domains protein 1 isoform 2; MYL1: Myosin light chain 1/3 skeletal muscle isoform; TNNI2: Troponin I fast skeletal muscle; CRYAB: Alpha-crystallin B chain; TNNC1: Troponin C. The higher and significant correlation coefficients are in bold.

## Data Availability

Data available on request.
